# Incidence and Risk Factors of Clostridium difficile Infection Among Adult Patients Admitted to the Inpatient Department of a Tertiary Care Hospital: A Hospital-Based Observational Study

**DOI:** 10.7759/cureus.75071

**Published:** 2024-12-03

**Authors:** Ishanvi Ishanvi, Shubhransu Patro, Vibha Sharma, Chikkam Sandeep, Smrutisree Mohapatra, Smaranita Sabat, Kumudini Panigrahi, Basanti Kumari Pathi

**Affiliations:** 1 Internal Medicine, Kalinga Institute of Medical Sciences, Bhubaneswar, IND; 2 General Medicine, Kalinga Institute of Medical Sciences, Bhubaneswar, IND; 3 Microbiology, Kalinga Institute of Medical Sciences, Bhubaneswar, IND; 4 Community Medicine, Institute of Medical Sciences & SUM Hospital, Bhubaneswar, IND

**Keywords:** antibiotic associated diarrhea, cd toxin a & b, clostridium difficile infection, laboratory diagnosis, stool elisa

## Abstract

Background

*Clostridium difficile* infection (CDI) is a significant healthcare concern, marked by its rising prevalence and associated morbidity and mortality. However, there is limited data on the epidemiology of CDI in the eastern region of India.

Objectives

The study aims to determine the incidence of CDI among adult patients admitted to the inpatient department of a tertiary care hospital and identify the risk factors associated with CDI.

Methodology

A prospective observational study was conducted at a tertiary care hospital between October 2022 and March 2023. Using universal sampling, 200 adult patients were included in the study. Stool samples were collected within 24 hours of admission and again on the day of discharge or after one week, whichever period was longer. Relevant clinical, demographic, and laboratory data were also collected. The stool samples were analyzed for *C. difficile* toxins A and B using an enzyme immunoassay. Statistical analysis was performed using the mean and independent t-test for continuous variables, while proportions and Chi-square or Fisher’s exact tests were applied for categorical variables.

Results

The incidence of CDI during the study period was 9%. The participants had a mean age of 46.49 ± 16.78 years, with a predominance of males (60%). Acute febrile illness was the most common diagnosis at admission (36%). The mean duration of hospitalization was significantly longer in patients who tested positive for *C. difficile* toxins via enzyme-linked immunosorbent assay compared to those who tested negative (8.0 ± 1.53 days vs. 3.75 ± 1.25 days, p < 0.001). Exposure to broad-spectrum antibiotics, particularly third-generation cephalosporins, was significantly associated with CDI development. Additionally, fecal leukocytes were detected in all (100%) patients who tested positive for *C. difficile* toxins.

Conclusions

This study offers important insights into the incidence and risk factors of CDI among adult patients in a tertiary care setting. The findings emphasize the need for the judicious use of broad-spectrum antibiotics to reduce the risk of CDI. Additionally, the detection of fecal leukocytes may serve as a valuable diagnostic marker for CDI in clinical practice.

## Introduction

*Clostridium difficile* is a spore-forming, Gram-positive, anaerobic bacillus ubiquitously found in the intestinal tracts of humans and animals, as well as in various environmental settings. Transmission occurs via the fecal-oral route through the ingestion of spores [1]. Healthcare environments often facilitate transmission through contaminated surfaces and the hands of healthcare workers [2]. *C. difficile* infection (CDI) is a leading cause of antibiotic-associated diarrhea, predominantly acquired as a nosocomial infection [3].

Since the late 20th century, CDI rates have risen markedly, particularly among individuals with recent or ongoing hospitalizations, with prevalence rates ranging from 3% to 26% [2]. In India, CDI has emerged as a significant cause of antibiotic-associated diarrhea, with reported prevalence rates of 5-20% in the western and central regions [4,5].

CDI is a toxin-mediated gastrointestinal disease caused by the exotoxins A and B, produced by *C. difficile*. The clinical spectrum ranges from mild, watery diarrhea and abdominal pain to severe conditions like life-threatening colitis [6]. Potential complications include toxic megacolon and intestinal perforation [7]. Risk factors for CDI include broad-spectrum antibiotic use, advanced age, prolonged hospitalization, and colonization by *C. difficile* [8].

CDI imposes a substantial burden on healthcare systems by prolonging hospital stays, increasing ICU admissions, and raising treatment costs. It also contributes significantly to morbidity and mortality, with all-cause mortality rates estimated between 13% and 30% [9]. While some patients recover with prompt treatment, others face challenges such as recurrent infections [2,9,10]. Effective prevention of CDI requires timely case detection and the implementation of comprehensive prevention strategies [10].

Case detection involves identifying clinical symptoms, particularly diarrhea defined as three or more loose or unformed stools within 24 hours [11], alongside diagnostic methods like stool culture, enzyme immunoassays, or PCR to detect *C. difficile* toxins or their genes [11,12]. Treatment typically involves antibiotics such as vancomycin, fidaxomicin, and metronidazole, although recurrence rates remain high, necessitating novel approaches like fecal microbiota transplantation for recurrent cases [13-16].

CDI represents a significant global health challenge, with increasing rates of community-acquired infections. Its impact on healthcare systems, particularly through heightened mortality, underscores the importance of understanding its burden in both hospital and community settings. However, studies on CDI in the eastern region of India remain limited, despite its unique sociodemographic and healthcare dynamics. The 2021 ICMR national surveillance study reported that less than 5% of its data originated from eastern India, highlighting significant knowledge gaps regarding CDI's epidemiology and burden in this region [10].

Determining the incidence of CDI and identifying the factors associated with infection among hospitalized patients can provide valuable insights for healthcare workers and stakeholders. These findings can guide the implementation of effective diagnostic methods, optimize therapeutic strategies, and strengthen infection control practices to mitigate the burden of CDI in healthcare settings.

Keeping these considerations in mind, the study was conducted with the aim of determining the incidence of CDI among adult patients admitted to the inpatient department of a tertiary care hospital and identifying the risk factors associated with CDI.

## Materials and methods

A prospective observational study was conducted over six months, from October 2022 to March 2023, after obtaining Institutional Ethics Committee approval (IEC approval no. KIIT/KIMS/IEC/1030/2022, dated August 25, 2022). The study was carried out at the Kalinga Institute of Medical Sciences in Bhubaneswar, India. All consecutively admitted patients aged over 18 years, regardless of clinical condition or gender, and who consented to participate, were enrolled. A total of 200 patients were included in the study.

Patient demographic data, including age and sex, as well as medical history, were collected via a case record form. This included information on underlying diseases such as irritable bowel syndrome, inflammatory bowel disease, type 2 diabetes mellitus, and other immune-related disorders. Clinical variables, including provisional diagnoses at admission, broad-spectrum antibiotic usage during hospitalization (such as fluoroquinolones, third- and fourth-generation cephalosporins, and carbapenems), and hospital-related variables for gastrointestinal symptoms like diarrhea, dysentery, and abdominal pain occurring after 72 hours of hospitalization, were also recorded. The duration of hospital stay was noted as well.

Two stool samples were collected from each patient: the first within 24 hours of admission and the second on the day of discharge or after one week, whichever was longer. Patients were provided with wide-mouth sterile containers with lids for stool collection and were instructed not to take any laxatives the night before collection. Fresh stool samples of 10-15 grams, either formed or unformed, were placed in leakproof containers and transported to the laboratory in ice packs to maintain a temperature of 4-8°C, then stored in a refrigerator for further testing.

Stool samples were processed in the microbiology laboratory. A routine microscopic examination was performed to detect fecal leukocytes or pus cells. The samples were tested for *C. difficile* toxins A and B using the enzyme-linked immunosorbent assay (ELISA) method in a multi-step process to confirm CDI cases.

ELISA kit and procedure

The Epitope Diagnostics ELISA kit for *C. difficile* Toxin A and B was used for toxin detection. The kit included all required reagents and instructions specific to toxin detection. The procedure was performed according to the manufacturer’s guidelines. At the end of the ELISA procedure, the absorbance (optical density, OD) of each microwell was measured using a Bio-Rad ELISA reader at 450 nm, with duplicate readings for each well.

Interpretation of results

The average OD value for each well was calculated. Results were interpreted according to the manufacturer's guidelines: a positive result was defined if the OD value exceeded the positive cutoff (0.168), negative if the OD was below the negative cutoff (0.138), and equivocal if the OD was between the two cutoffs (0.139-0.167). For equivocal results, the test was repeated with a different sample.

Clinical and laboratory data were entered into Microsoft Excel. Statistical analysis was performed using IBM SPSS Statistics for Windows, Version 26.0 (Released 2019; IBM Corp., Armonk, NY, USA). Descriptive statistics such as mean, standard deviation, and median were calculated for continuous variables. Proportions were determined for categorical variables. The Mann-Whitney U test was used to assess associations between continuous variables, while the chi-square or Fisher’s exact test was used for categorical variables. A p-value of less than 0.05 was considered statistically significant.

## Results

The present study enrolled a total of 200 patients admitted to the medicine wards of a tertiary care hospital. The incidence of CDI during the study period was found to be 9% (18/200). The median age of the participants was 45.5 years, with a range from 18 to 80 years. Male patients (60%) outnumbered female patients (40%). A total of 38 patients (19%) had a history of prior hospitalization, and 22% had underlying diseases. The median duration of hospitalization among participants was four days. Broad-spectrum antibiotic exposure during hospitalization was noted in 60% of patients.

After 72 hours of hospitalization, 21% of the participants developed loose stools. At the time of admission (when the first stool samples were collected), none of the samples showed the presence of pus cells on routine microscopic examination or *C. difficile *toxin on the ELISA test. However, stool samples collected at discharge or after one week (second stool sample) tested positive for both stool pus cells and *C. difficile* toxins by ELISA in 9% of cases. Table 1 provides the sociodemographic and clinical parameters of the study participants.

**Table 1:** Sociodemographic and clinical parameters of study participants IPD, inpatient department

Sociodemographic and clinical variables	Number (percentage)
Age	
Mean age (years ± SD)	46.49 ± 16.74
Median age (years)	45.5
Sex	
Female	80 (40%)
Male	120 (60%)
Recent hospitalization (within two months)	38 (19%)
Underlying diseases present	44 (22%)
Duration of hospital stay in days	
Mean duration of hospital stay in days	4.14 ± 1.76
Median duration of hospital stay in days	3
Broad-spectrum antibiotic exposure in IPD	120 (60%)
Development of GI symptoms (after 72 hours of hospitalization)	42 (21%)
Stool samples collected on the day of admission	
Presence of pus cells in stool routine microscopy	0
*Clostridium difficile* toxin A and B ELISA positive	0
Stool samples collected on the day of discharge	
Presence of pus cells in stool routine microscopy	18 (9%)
*C. difficile *toxin A and B ELISA test positive	18 (9%)

**Figure 1:**
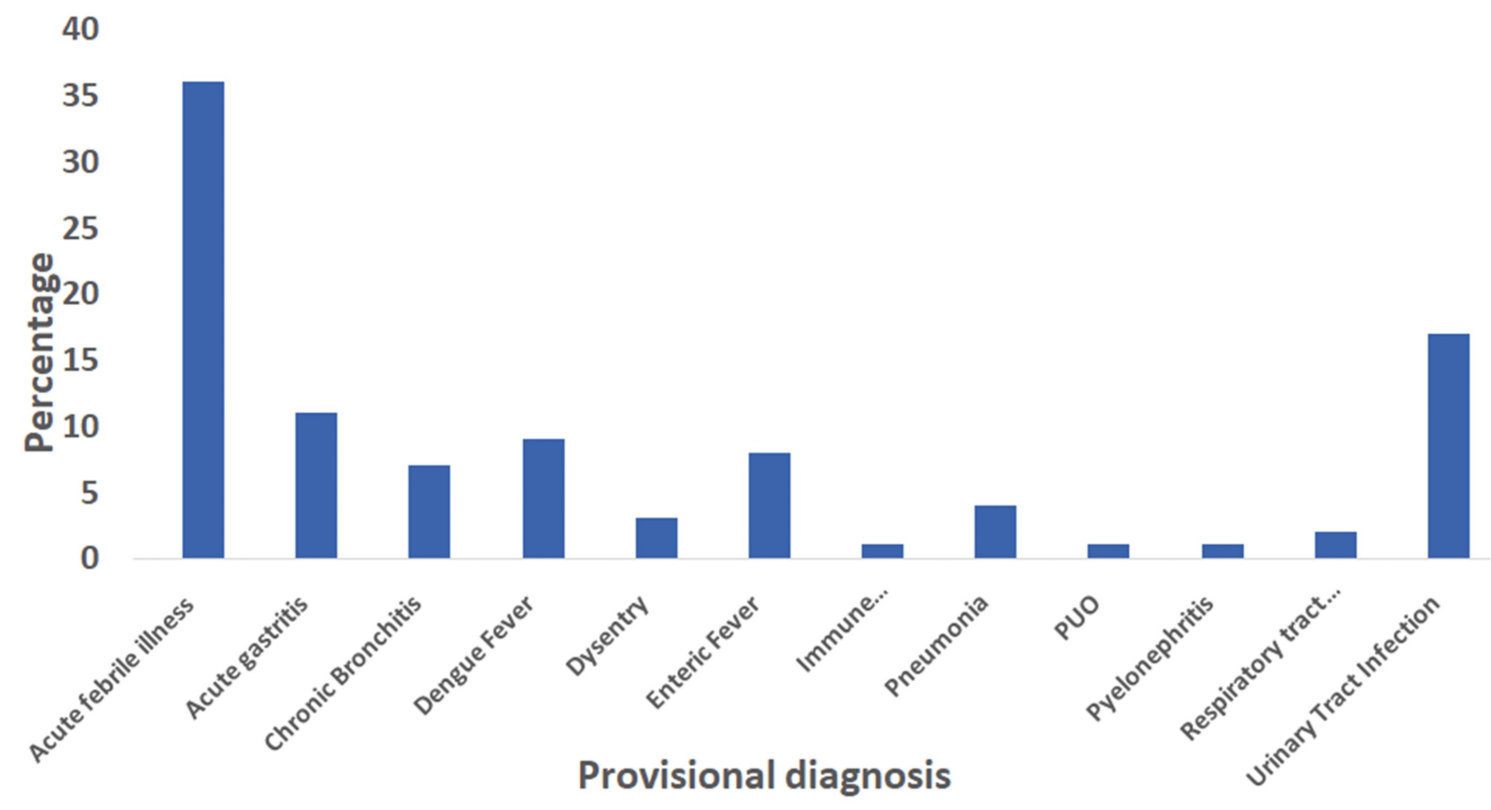
Provisional diagnosis of the study participants at the time of admission

Figure 1 shows that among the 200 admitted patients, acute febrile illness was the most common provisional diagnosis, accounting for 36% of cases, followed by urinary tract infection at 17%.

Table 2 presents the sociodemographic and clinical profile of the *C. difficile*-infected cases. The mean age of the *C. difficile *toxin-positive patients was 50.77 ± 16.12 years, with a median age of 49 years. CDI was detected in 10% (12/120) of male patients and 7.5% (6/80) of female patients. Among participants with and without a history of prior hospitalization, CDI was found in 4/38 (10.5%) and 14/162 (8.6%), respectively. Fourteen of the 18 toxin-positive cases had no underlying comorbidities. There was no significant association between CDI-positive cases and prior hospitalization or underlying comorbidities.

**Table 2:** Sociodemographic and clinical profile of Clostridium difficile-infected cases The statistical analyses performed include the Chi-square test, *Mann-Whitney U test, and **Fisher’s exact test to determine the associations between variables.

Variables	*Clostridium difficile* toxin ELISA positive cases (n = 18)	*C. difficile*toxin ELISA negative cases (n = 182)	p-value
Mean age	50.77 ± 16.12	46.06 ± 16.78	0.256*
Median age (years)	49	45	0.24*
Male	12 (10%)	108 (90%)	0.545
Prior hospitalization	4 (10.5%)	34 (89.5%)	0.753**
Concomitant diseases	4 (9.1%)	40 (90.9%)	0.981**
In-hospital broad-spectrum antibiotic exposure	18 (15%)	102 (85%)	<0.001**
History of diarrhea	12 (28.6%)	30 (71.4%)	<0.001**
Median duration of hospital stay (days)	8	3	<0.001*
Stool pus cells at the time of discharge	18 (100%)	0	<0.001**

The median duration of hospital stay was significantly longer in ELISA-positive cases (eight days) compared to ELISA-negative cases (three days). All CDI cases (100%) were exposed to broad-spectrum antibiotics during hospitalization. After 72 hours of hospitalization, 42/200 patients developed loose stools, 28.6% of whom were *C. difficile* toxin ELISA positive. Stool pus cells were detected in all CDI-positive cases. The development of CDI was significantly associated (p < 0.001) with the mean duration of hospitalization, exposure to broad-spectrum antibiotics, the development of gastrointestinal symptoms, and the presence of stool pus cells.

**Figure 2:**
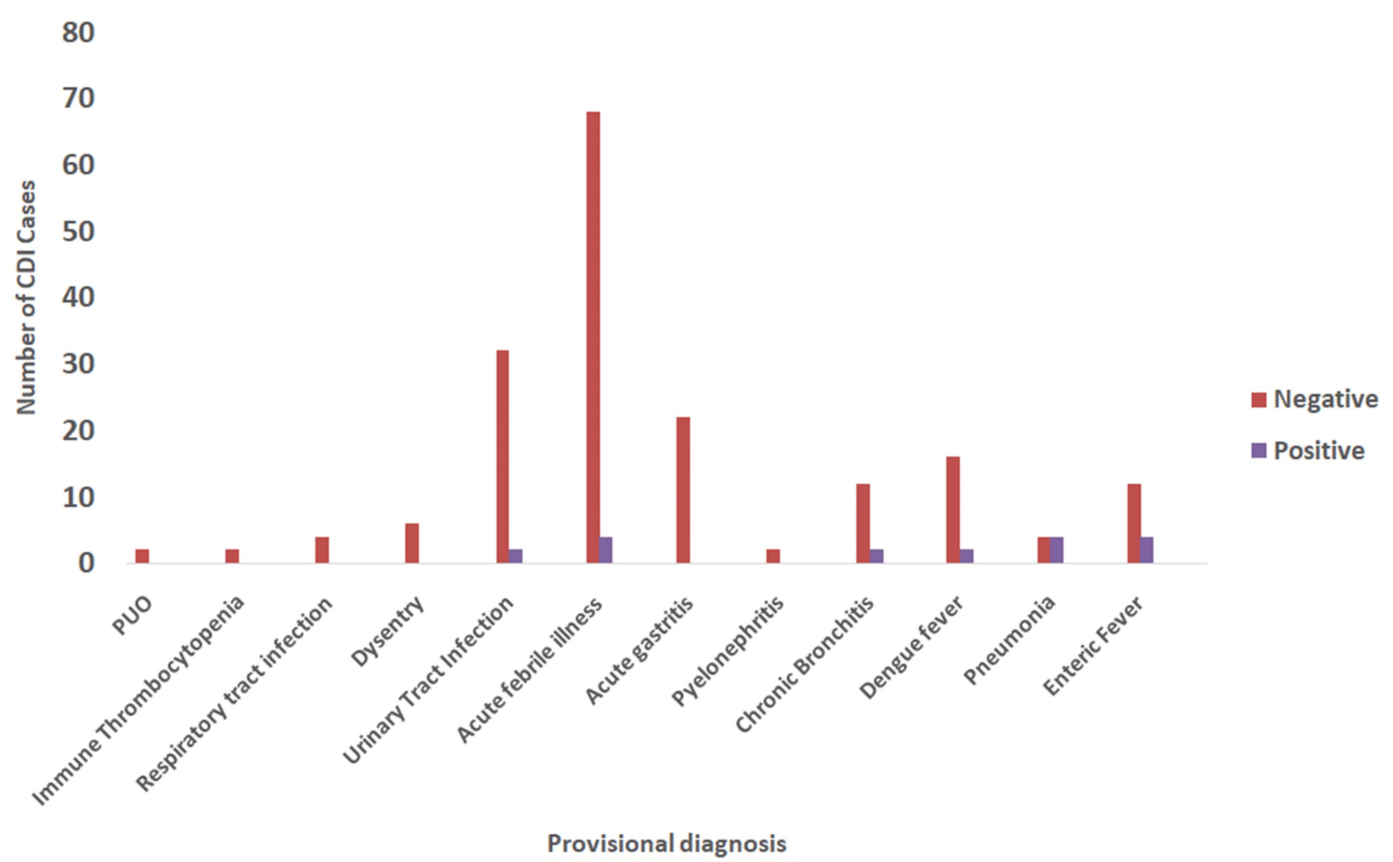
Distribution of Clostridium difficile toxin-positive cases across different diseases PUO, pyrexia of unknown origin

Figure 2 shows that CDI cases were most commonly detected among patients with acute febrile illness, pneumonia, and enteric fever.

**Figure 3:**
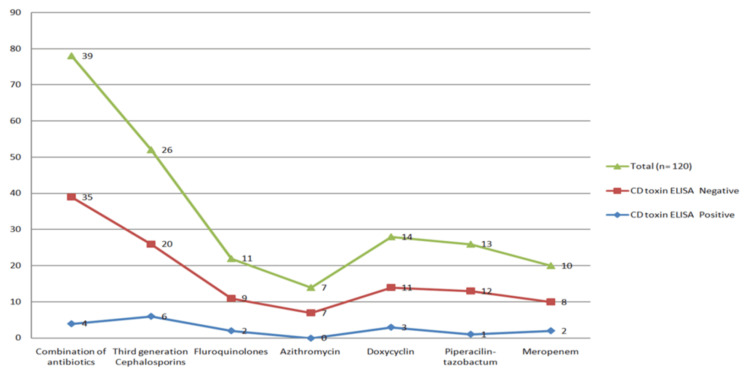
Frequency of antibiotic exposure (in numbers) among Clostridium difficile toxin-positive and toxin-negative cases

In Figure 3, the frequency of antibiotic use among *C. difficile *toxin ELISA positive and negative cases is presented. Among the 200 study participants, the majority (32%) were treated with a combination of various antibiotics, followed by third-generation cephalosporins. Of the 18 CDI cases, the majority (six cases) were treated exclusively with third-generation cephalosporins, followed by a combination of antibiotics (four cases).

## Discussion

CDI is a significant healthcare concern, primarily due to its association with antibiotic use and nosocomial transmission. This study aimed to investigate the incidence of CDI among patients attending a tertiary care teaching hospital and explore the associated risk factors and clinical characteristics.

Our findings revealed that the incidence of CDI among hospitalized patients during the study period was 9%, aligning with previous studies reporting incidence rates ranging from 3% to 29% [4,5,17-19]. Notably, this rate was slightly higher than that reported by Ozaki et al. [20], but consistent with studies by Chaudhry et al., which reported a 6% incidence rate [21]. Interestingly, studies from India have shown considerable variation in CDI prevalence, with some reporting rates as high as 25-30% among hospitalized patients with diarrhea. In contrast, Segar et al. reported a prevalence rate of 4% in their study [22]. These differences can be attributed to variations in population characteristics, diagnostic methods, and hospital settings.

The relatively low prevalence observed in our study could be due to several factors, including selective prescribing of probiotics to patients on prolonged and broad-spectrum antibiotic therapy, stringent surveillance with an improved antibiotic policy, and the active involvement of the hospital infection control team. Additionally, we did not include ICU-admitted patients in our study, who are typically exposed to higher antibiotic use and have longer hospital stays, increasing the likelihood of CDI occurrence.

Although age was not found to be a significant factor for CDI development in this study, the mean age of the *C. difficile* toxin-ELISA positive cases was numerically higher (50.77 ± 16.12 years) than that of the negative cases. This finding is consistent with previous studies by Vishwanath et al. [18] and Segar et al. [22], which reported a higher prevalence of CDI among older patients. Advanced age (≥60 years) is a recognized risk factor for CDI, likely due to increased healthcare exposure, antibiotic use, and the presence of underlying comorbidities. However, this observation was not statistically significant in our study and should be interpreted with caution.

Regarding gender distribution, our study showed a higher proportion of *C. difficile *toxin-positive cases among males (60%), consistent with findings by Chaudhry et al. [21]. This finding contrasts with reports by Vaishnavi et al., suggesting variability in CDI gender distribution across different studies [23]. Gender differences in CDI incidence may result from biological factors such as differences in immune responses and sociocultural factors like variations in healthcare-seeking behavior or antibiotic exposure. Further studies are needed to understand the factors contributing to gender disparities in CDI prevalence.

In this study, cases of acute febrile illness, pneumonia, and enteric fever were associated with CDI development, likely due to the widespread use of broad-spectrum antibiotics and prolonged hospital stays. Remarkably, the majority of toxin-positive cases in our study lacked underlying comorbidities, indicating that CDI can affect individuals regardless of their health status. The mean duration of hospitalization among ELISA-positive cases was significantly longer (8.0 ± 1.53 days) than that of ELISA-negative cases (3.75 ± 1.25 days). The strong association between hospitalization duration and CDI positivity highlights the role of prolonged hospital stays as a risk factor for CDI acquisition, which is consistent with research conducted by Choudhury et al., emphasizing the importance of hospital infection control measures and antimicrobial stewardship programs [21].

Our study highlighted antibiotic exposure as a critical risk factor for CDI, as all toxin-positive cases (100%) were exposed to broad-spectrum antibiotics during hospitalization. Third-generation cephalosporins were the most frequently implicated antibiotic class, in line with findings from studies showing an association between cephalosporin use and CDI incidence [19]. This reinforces the need for cautious and judicious antibiotic prescribing practices to mitigate CDI risk in hospital settings.

The development of gastrointestinal symptoms after 72 hours of hospitalization was significantly associated with CDI in our study, consistent with findings from previous research [19]. Additionally, the presence of fecal leukocytes in stool samples from CDI-positive patients indicated an inflammatory response, which is commonly associated with CDI. However, studies by Reddymasu et al. have noted the poor predictability and low sensitivity of fecal leukocyte counts for CDI detection [24].

Limitations

Our study had a few limitations. The study duration was short, and the sample size was relatively small. Only ELISA was employed for CDI detection, and we only included patients from indoor wards. Including patients from all hospital wards and ICUs could provide a more comprehensive analysis of CDI trends. Furthermore, molecular testing, such as PCR for toxin gene detection, is more sensitive and specific, and using this method might yield a more accurate CDI rate.

## Conclusions

The present study provides valuable insights into the incidence rate, risk factors, and clinical characteristics of CDI among hospitalized patients. The incidence of CDI was found in one-tenth of the study population. Patients admitted with provisional diagnoses of acute febrile illnesses and pneumonia were more likely to develop CDI. Significant factors for CDI development included the use of third-generation cephalosporins and longer hospitalization durations. These findings suggest that targeted interventions are needed to reduce CDI incidence and associated morbidity and mortality. The results emphasize the importance of implementing effective infection control measures, including hand hygiene, contact precautions, surface disinfection, antimicrobial stewardship programs, and diagnostic strategies to mitigate the CDI burden in healthcare settings. Further research is needed to explore CDI diagnostics, risk factors, pathogenesis, and community-acquired CDI to develop targeted diagnostic, therapeutic, and preventive strategies.
